# Intermediate levels of asymptomatic transmission can lead to the highest epidemic fatalities

**DOI:** 10.1093/pnasnexus/pgad106

**Published:** 2023-03-29

**Authors:** Sang Woo Park, Jonathan Dushoff, Bryan T Grenfell, Joshua S Weitz

**Affiliations:** Department of Ecology and Evolutionary Biology, Princeton University, Princeton, NJ, USA; Department of Biology, McMaster University, Hamilton, ON, Canada; Department of Mathematics and Statistics, McMaster University, Hamilton, ON, Canada; M. G. DeGroote Institute for Infectious Disease Research, McMaster University, Hamilton, ON, Canada; Department of Ecology and Evolutionary Biology, Princeton University, Princeton, NJ, USA; Princeton School of Public and International Affairs, Princeton University, Princeton, NJ, USA; School of Biological Sciences, Georgia Institute of Technology, Atlanta, GA, USA; School of Physics, Georgia Institute of Technology, Atlanta, GA, USA; Institut de Biologie, École Normale Supérieure, Paris, France

## Abstract

Asymptomatic infections have hampered the ability to characterize and prevent the transmission of SARS-CoV-2 throughout the pandemic. Although asymptomatic infections reduce severity at the individual level, they can make population-level outcomes worse if asymptomatic individuals—unaware they are infected—transmit more than symptomatic individuals. Using an epidemic model, we show that intermediate levels of asymptomatic infection lead to the highest levels of epidemic fatalities when the decrease in symptomatic transmission, due either to individual behavior or mitigation efforts, is strong. We generalize this result to include presymptomatic transmission, showing that intermediate levels of nonsymptomatic transmission lead to the highest levels of fatalities. Finally, we extend our framework to illustrate how the intersection of asymptomatic spread and immunity profiles determine epidemic trajectories, including population-level severity, of future variants. In particular, when immunity provides protection against symptoms, but not against infections or deaths, epidemic trajectories can have faster growth rates and higher peaks, leading to more total deaths. Conversely, even modest levels of protection against infection can mitigate the population-level effects of asymptomatic spread.

**Significance Statement:**

During an epidemic, asymptomatically infected individuals may avoid severe outcomes but can still transmit, potentially leading to severe outcomes in others, including fatalities. This manuscript analyzes the population-level effects of asymptomatic spread in the presence of strong transmission reduction among symptomatic individuals due to behavioral change and interventions. Theory and simulations reveal that when reduction is strong the number of infections increases with asymptomatic proportion, while fatalities peak at intermediate levels. The same framework also shows how milder variants at the individual level can potentially lead to worse outcomes for the population—of relevance to ongoing efforts to explore the interplay between behavior, immunity, and viral variants.

## Introduction

SARS-CoV-2 has had devastating effects at the population level. However, many individuals experienced mild cases, making it harder to estimate the magnitude of spread and fatality rate (1). The ratio of fatalities to documented *cases* (the case-fatality rate, CFR) is typically between 1 and 4%, varying across population because of testing patterns, treatment practice, case definitions, and other factors (2–4). But many infections are never documented; the ratio of fatalities to total *infections* (the infection fatality rate, IFR) has been estimated to be closer to 0.5–1% for prevaccinated populations whose demographics are similar to those of the United States (5). This means that more than 99% of individuals infected with COVID-19 will survive. Moreover, at least half of the infections are sufficiently mild that they could be classified as subclinical.

**Fig. 1.**
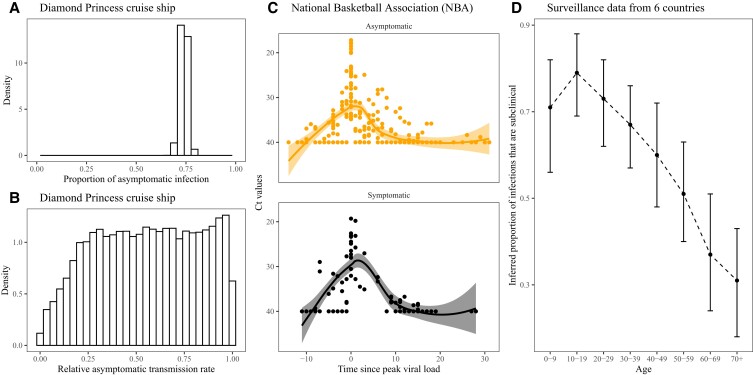
Asymptomatic transmissibility of SARS-CoV-2. A) Posterior estimates of the proportion of asymptomatic infections from the Diamond Princess cruise ship (12). B) Posterior estimates of the ratio $\theta_{a}$ of the transmission rates between asymptomatic and symptomatic individuals from the Diamond Princess Cruise Ship (12). Symptomatic individuals were assumed to transmit at rate β(t) for an average of 2.9 days, followed by a presymptomatic stage with an average of 2.1 days. Asymptomatic individuals were assumed to transmit at rate $\theta_{a}\beta (t)$ for an average of 5 days. Both estimates are publicly available with further details in (12). C) Viral load trajectory data from players, staff, and vendors of the National Basketball Association (NBA). Points represent each Ct measurement. Lines and shaded areas show default LOESS fits from ggplot2 (13). Data are publicly available in (14). D) Inferred proportion of infections that are subclinical for each age group using surveillance data from six countries (China, Italy, Japan, Singapore, South Korea, and Canada) (15).

It is now well established that asymptomatic individuals can transmit SARS-CoV-2 infections (6–10), but asymptomatic cases are increasingly shaped by prior immunity (whether through infection, vaccination, or both). In contrast, early in the pandemic, a COVID-19 outbreak on the Diamond Princess cruise ship played a critical role in understanding the role of asymptomatic infections in the spread of SARS-CoV-2 from and to immunologically naive individuals; the outbreak occurred among 3711 passengers and crew, of whom 634 individuals tested positive by 20 February 2020 (11). It has been estimated that 75% (95% CI: 70–78%) of all infections on the cruise ship were asymptomatic (Fig. 1A) with about half of total infections undetected (12). The relative transmission rate of asymptomatic individuals aboard the Diamond Princess was not well constrained by the analysis, but low relative transmission rate (below 25%) by asymptomatic individuals was ruled out because it required unrealistically high transmissibility for symptomatic individuals (Fig. 1B).

Modeling studies have typically assumed that transmissibility is lower for asymptomatic than for symptomatic individuals; assumptions have ranged from 10 to 100% (16, 17). Similarities in viral load trajectories of asymptomatic and symptomatic individuals provide indirect support for the transmissibility of asymptomatic individuals (Fig. 1C, (14)); however, differences between inferred *total* viral load from Ct values and *infectious* viral load add uncertainty (18). The likely role of symptoms in transmission further contributes to this uncertainty. Coughing and sneezing can help deliver virus-containing droplets. However, the prevalence of presymptomatic and asymptomatic transmission of SARS-CoV-2 suggests that speech droplets can also be an important mode of transmission (19).

We note also that asymptomaticity can change across outbreak settings (20). For example, during the early pandemic, Davies *et al.*’s analyses of surveillance data across six countries revealed that older individuals were less likely to have subclinical infections (Fig. 1D), providing indirect evidence for heterogeneity in asymptomaticity (15). Differences in contact rates between age classes further contribute to the heterogeneity in asymptomatic transmissibility. For now, we primarily focus on a homogeneous population and return to the age effect in discussing our model-based findings.

Despite quantitative uncertainties in asymptomatic transmissibility, individuals infected asymptomatically with SARS-CoV-2 can still transmit to others. This means that the presence of asymptomatic infections may have countervailing effects at the population level. On one hand, an asymptomatic infection means that the individual infected avoids hospitalization and death. On the other hand, asymptomatic infections are less likely to be detected (21, 22), meaning that asymptomatic individuals are less likely to take precautions and relatively more likely to infect others; asymptomatic SARS-CoV-2 infections present additional challenges to managing overall disease burden due to the possibility of long COVID (23).

In this manuscript, we explore the effects of asymptomatic infection and transmission on disease severity at the population level. In doing so, we assume that symptomatic individuals reduce their transmission, reflecting changes in behavior (e.g., self-isolation after symptom onset) and/or nonpharmaceutical intervention measures. Under this assumption, we show that a high proportion of asymptomatic infections could paradoxically make population-level outcomes worse than if SARS-CoV-2 was more dangerous at the individual level. We further extend our framework to understand the interaction between immunity against symptomatic infections on the dynamics of emerging variants and explore mechanisms by which milder variants at the individual level can nonetheless lead to similar or worse population-level outcomes.

## Results and discussion

**Fig. 2.**
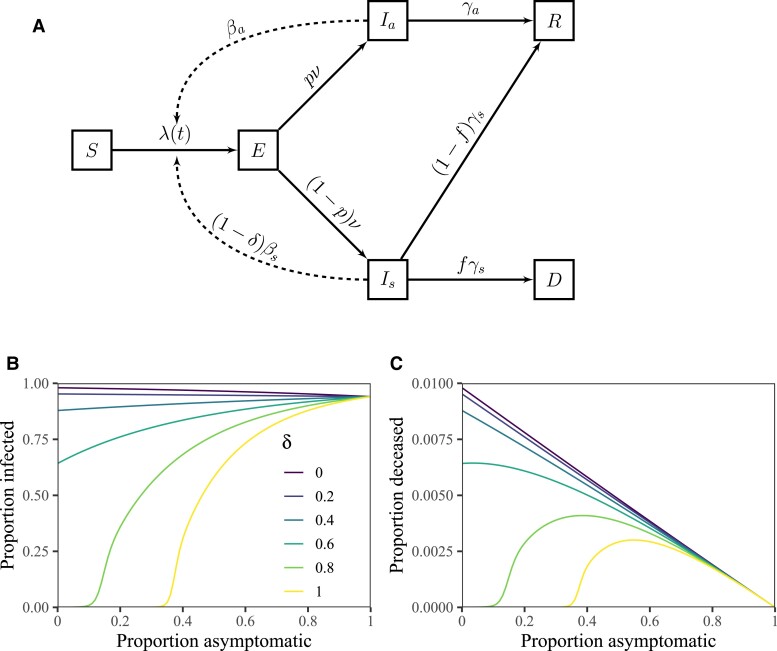
Schematic diagram and simulations of a model with asymptomatic transmission and symptom-responsive transmission reduction. A) *S* represents susceptible individuals; *E* represents exposed individuals; $I_{a}$ represents asymptomatic individuals; $I_{s}$ represents symptomatic individuals; *R* represents recovered individuals; and *D* represents deceased individuals. See Methods for model details. B) Total infections as a function of the proportion of asymptomatic infections *p* across a wide range scenarios for δ. C) Total deaths as a function of the proportion of asymptomatic infections *p* across a wide range scenarios for δ. We simulate the model for 365 days, assuming $\beta_{s}=0.8/\mathrm{day}$, $\beta_{a}=0.75\beta_{s}$, ν=0.5/day, $\gamma_{s}=\gamma_{a}=0.2/\mathrm{day}$, and f=0.01, and an initial exposed proportion of $10^{-4}$. See Materials and Methods for model details and Supplementary Table S1 for parameter descriptions and values.

We propose an epidemic model in which infected individuals can be asymptomatic or symptomatic, with probabilities *p* and 1−p, respectively (Fig. 2A). Asymptomatic individuals always recover, whereas a fraction *f* of symptomatic individuals die. Asymptomatic and symptomatic individuals can also have different infection characteristics, including their transmission rates ($\beta_{a}$ and $\beta_{s}$) and removal rates ($\gamma_{a}$ and $\gamma_{s}$). Our key assumption is that symptomatic individuals take greater precautions than do asymptomatic individuals (e.g., reducing contacts or increasing mask-wearing) and therefore reduce their transmission rate by a fraction δ; the parameter δ may also capture intervention measures that target symptomatic individuals, such as symptom-based isolation. We note that intervention measures that target asymptomatic infections would reduce the effective value of δ—for example, frequent testing and isolation may effectively increase the removal rate $\gamma_{a}$ of asymptomatic individuals. For our main simulations, we assume that asymptomatic individuals have a lower reproduction number—this is implemented via lower transmission rates for asymptomatic individuals ($\beta_{a}=0.75\beta_{s}$) and equal removal rates ($\gamma_{a}=\gamma_{s}$). We then evaluate the effects on population-level mortality of changing the asymptomatic proportion *p* while holding the fatality rate *for symptomatic cases*, *f*, constant (the population-level IFR (1−p)f thus decreases as *p* increases).

Fig. 2B and C shows simulated epidemic outcomes using parameters similar to those of the originating strain of SARS-CoV-2 (Table S1), without any mitigation other than that individuals who are symptomatic reduce their transmission rate by δ. For this model, the basic reproduction number is given by:


(1) $$R_{0}=(1-p)(1-\delta )R_{s}+pR_{a},$$

where $R_{s}=\beta_{s}/\gamma_{s}$ and $R_{a}=\beta_{a}/\gamma_{a}$ represent the reproduction numbers of asymptomatic and symptomatic individuals (i.e., the average number of secondary infections caused by asymptomatic and symptomatic individuals); therefore, in the absence of the behavioral effect (δ=0), the final size decreases with the asymptomatic proportion *p* because more symptomatic infections leads to a higher basic reproduction number.

This relationship changes as δ increases. In particular, when $\delta >1-R_{a}/R_{s}$ (in this case, δ>0.25), the basic reproduction number (and thus epidemic size) increases with *p* because the effective symptomatic reproductive number (including behavioral response) is less than that the asymptomatic reproductive number. When $\delta >1-1/R_{s}$( and $R_{a}>1$), we can find a critical asymptomatic proportion, $p_{c}$:


(2) $$p_{c}=\frac{1-(1-\delta )R_{s}}{R_{a}-(1-\delta )R_{s}}$$

such that an outbreak will occur exactly when $p>p_{c}$ (see threshold effects for large values of δ in Fig. 2B).

When behavioral protection is high, the effect of asymptomatic proportion on fatalities shows countervailing effects of individual-level protection and population-level risk (Fig. 2C). For high values of δ, the peak fatality occurs at intermediate levels of asymptomatic spread: although fewer individuals die per infection for higher values of *p*, the increase in total infections still leads to an increase in total fatalities. In contrast, when δ is small enough that $(1-\delta )R_{s}\geq R_{a}$ (in this case, δ<0.25), both the number of infections and the IFR ((1−p)f) decrease with increasing *p*. We note that these results are robust to uncertainties in asymptomatic transmission rate. In Supplementary Figure S1, we perform the same analysis while varying the ratio between $R_{a}$ and $R_{s}$ between 0.25 and 1. In this case, qualitative predictions are robust when $R_{a}\geq 0.5R_{s}$—when $R_{a}=0.25R_{s}$ and δ is large then $R_{0}<1$ and the epidemic does not take off. In Supplementary Figure S2, we perform the same sensitivity analysis as Supplementary Figure S1 while fixing $R_{0}=4$ when p=0.5 and δ=0 to prevent the epidemic from dying out. In this case, we find that intermediate values of asymptomaticity lead to the highest epidemic fatalities at high δ values across all ranges of $R_{a}/R_{s}$ we consider.

We can ask whether the high values of δ required for the nonlinear effects of asymptomaticity on deaths are realistic. For this particular model, it does not make biological sense for δ to be greater than the amount of postsymptomatic transmission, because presymptomatic transmission is implicitly included in the $I_{s}$ compartment. While several studies have estimated the proportion of presymptomatic transmission to be around 30–60% for the SARS-CoV-2 wildtype strain, many of these were likely affected by intervention and behavioral effects, as they were conducted after SARS-CoV-2 awareness became widespread (24). Instead, (25) recently estimated that the proportion of presymptomatic transmission could have been as low as 20% (95%CI: 6–32%) during the first few weeks of the pandemic when the pandemic-awareness and intervention measures were minimal. There are two implications of this updated estimate—first, a low proportion of presymptomatic transmission makes high δ values at least somewhat more likely during the initial pandemic phase; and second, substantial levels of behavioral effects (δ>0) may have been present early in the pandemic (to reduce the proportion of symptomatic transmission from 80% to as low as 40%).

To further address the uncertainty in the values of δ, we extend our model to include both presymptomatic and asymptomatic transmission. To do so, we reparameterize the model based on the relative importance of *nonsymptomatic* transmission, rather than on the proportion of asymptomatic cases. We then use $\delta_{s}$ to capture the decrease in transmission only after symptom onset—this means that $\delta_{s}$ is now independent of the amount of presymptomatic transmission. We then fix the reproduction number of symptomatic individuals and calculate fatalities at the population level as a function of the proportion of total nonsymptomatic transmission and the proportion of nonsymptomatic transmission that is caused by presymptomatic transmission (see Materials and Methods for model details and Supplementary Table S2 for parameter descriptions and values).

Using the generalized nonsymptomatic transmission model, we find a wide variety of scenarios for which peak fatalities occur at intermediate levels of nonsymptomatic transmission in the presence of moderate to strong behavioral effects, $\delta_{s}>0.6$ (Supplementary Fig. S3; Table S2). For example, when 40% of nonsymptomatic transmission is caused by presymptomatic transmission, a 60% reduction in transmission after symptom onset is sufficient to drive the nonlinear effect of nonsymptomatic transmission on epidemic fatality (peaking at around 10% nonsymptomatic transmission). One exception is the extreme case in which all nonsymptomatic transmission is caused by presymptomatic transmission (i.e., there is no asymptomatic transmission); in this case, total infections and fatalities are maximized when all transmission is caused by presymptomatic transmission. While 60% reduction in transmission after symptom onset ($\delta_{s}=0.6$) is still high, it is plausible given behavioral and policy responses. For example, (26) estimated that up to ∼64% reduction in transmission would be possible under self-isolation and self-quarantine as well as manual contact tracing (and as low as ∼47% reduction using digital contact tracing without manual tracing). Given that the majority of isolation and tracing measures likely target symptomatic transmission, the amount of reduction in symptomatic transmission would be similar to these values, providing indirect support for the feasibility of high $\delta_{s}$ values. Hereafter, we focus on asymptomatic infections for simplicity, but our conclusions have implications for the more general case of nonsymptomatic transmission. We return to the discussion of values of δ later in the Discussion section.

**Fig. 3.**
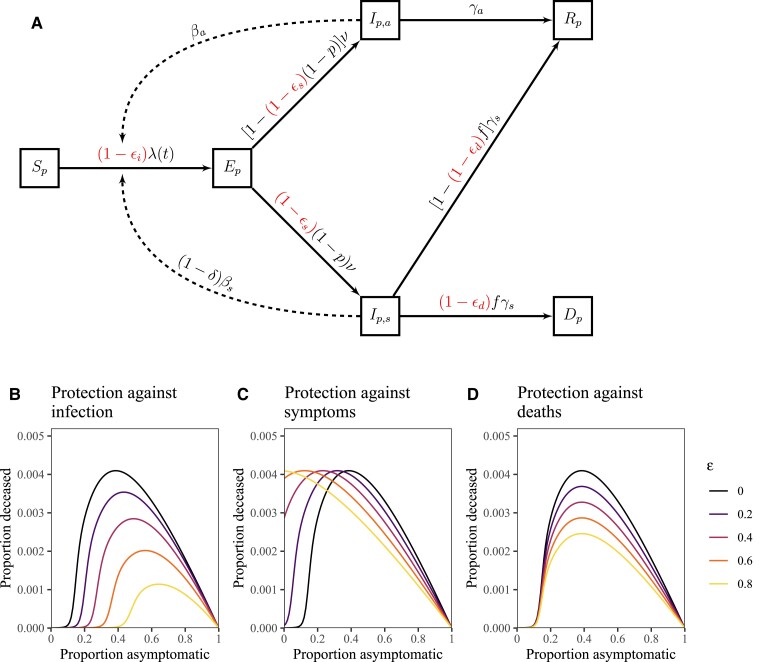
Schematic diagram and simulations of a model with symptom-responsive transmission reduction and immunity. A) The subscript *p* represents protected individuals. Immunity may provide protection against infection, symptoms, or deaths. The dynamics of immunologically naive individuals are described in Fig. 2. B–D) Total deaths as a function of the proportion of asymptomatic infections *p* across a wide range scenarios for protection against infection $\epsilon_{i}$ (B), symptoms $\epsilon_{s}$ (C), and deaths $\epsilon_{d}$ (D). We simulate the model for 365 days, assuming $\beta_{s}=0.8/\mathrm{day}$, $\beta_{a}=0.75\beta_{s}$, ν=0.5/day, $\gamma_{s}=\gamma_{a}=0.2/\mathrm{day}$, f=0.01, and δ=0.8. The initial infected proportion is $10^{-4}$. See Materials and Methods for model details and Supplementary Table S3 for parameter descriptions and values.

We now apply our framework to understand the impact of immunity on total fatalities at the population scale by dividing the population into two groups: immunologically naive and protected. For simplicity, we do not distinguish whether the immunity is derived from natural infections or vaccines. The dynamics of immunologically naive individuals are equivalent to our original model (Fig. 2). The dynamics of protected individuals include three additional parameters, which characterize the amount of protection against infection $\epsilon_{i}$, symptoms (given infection) $\epsilon_{s}$, and deaths (given symptoms) $\epsilon_{d}$ (Fig. 3). For simplicity, we assume that the population is split in half (50% naive and 50% protected) and mixes homogeneously. We also do not consider the separate effect of immunity on transmission (beyond the effect on infection). In other words, we assume that asymptomatic infections in protected and unprotected people have the same reproduction numbers (and likewise for the symptomatic infections). In practice, both asymptomatic and symptomatic infections in protected people are less likely to transmit than their unprotected counterparts (27): asymptomatic infections in protected people may indicate limited viral replication or even immune boosting, in which case an exposed individual may successfully fight off the pathogen early in infection before it can be transmitted; and symptomatic infections in protected people may reflect a strong immune response (rather than high viral load), in which case symptomaticity can be a poor proxy for transmission. We assume a relatively strong behavioral effect δ=0.8 for illustration (Table S3).

We consider each protection effect—$\epsilon_{i}$, $\epsilon_{s}$, and $\epsilon_{d}$—separately and consider joint effects later on. The impact of protection against infection $\epsilon_{i}$ is analogous to changing $R_{0}$ in the original model: as immunity provides stronger protection against infection (higher $\epsilon_{i}$), the number of deaths decreases and a higher asymptomatic fraction *p* is required for the infection to spread (Fig. 3B). We note that protection against infection scales the fatality curve nonlinearly, reflecting the nonlinear relationship between $R_{0}$ and the final size of the outbreak. The impact of protection against symptoms $\epsilon_{s}$ is equivalent to changing the asymptomatic fraction *p* for the protected population because protected individuals are less likely to develop symptoms: the peaks of the fatality curves move to lower values of *p* as we increase the degree of protection $\epsilon_{s}$ (Fig. 3C). Therefore, for low values of *p*, protection against symptoms can increase the total number of fatalities at the population level by increasing the proportion (and number) of asymptomatic individuals, who can readily transmit infections to other individuals. This also means that the critical asymptomatic proportion decreases, allowing more dangerous infections (with lower *p*) to invade, which would not have been able to spread in an otherwise immunologically naive population. We note that the equivalence between protection against symptoms $\epsilon_{s}$ and fraction asymptomatic *p* relies on our assumption that immunity does not provide protection against transmission. Protection against deaths $\epsilon_{d}$ directly modulates the fatality rate for symptomatic cases and therefore linearly scales the fatality curves (Fig. 3D).

**Fig. 4.**
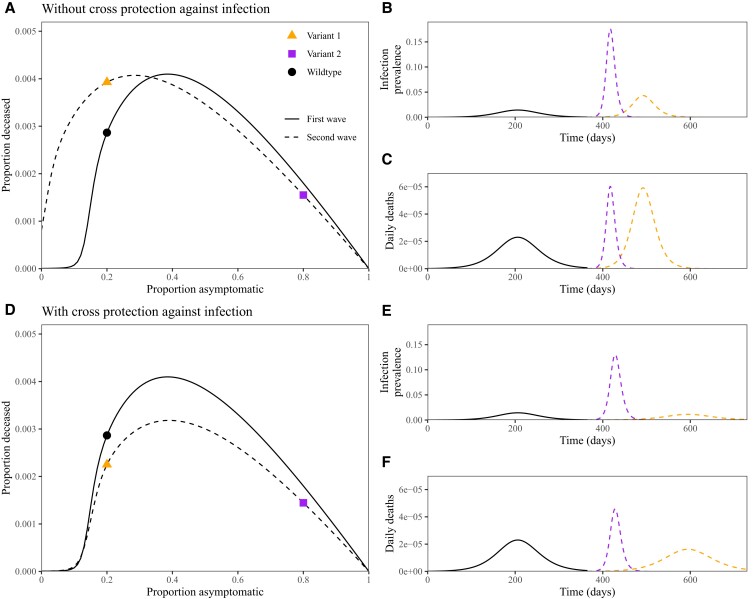
Dynamics of invading variants under symptom-responsive transmission reduction and immunity. A,D) Asymptomaticity–fatality curves for the first (solid lines) and second waves (dashed lines). Points represent specific scenarios we assume for the first and second waves. Fatality curves for the first wave are calculated by simulating an epidemic for 1 year using parameters from Fig. 2 with δ=0.8. Fatality curves for the second wave are calculated by first simulating the first wave assuming p=0.2 for 1 year to calculate the proportion immune and then simulating the extended model presented in Fig. 3 for *either* a milder (p=0.8) or an equally severe (p=0.2) variant. B,E). Dynamics of infection prevalence for the wildtype variant (black, solid line) and milder (purple, dashed line with earlier peak) and similar (orange, dashed line with later peak) invading variants. C,F) Dynamics of daily deaths for the wildtype variant (black, solid line) and two possible invading variants (colored, dashed line).

Finally, we use our framework to understand the impact of behavioral effects on invading variants (Fig. 4). We first simulate the dynamics of a wildtype variant for 1 year using our base model with parameters as in Fig. 2. We then simulate a new variant invading a partially immune population using our extended model (Fig. 3A), where the immunity is solely derived from natural infections caused by the wildtype variant in the first year. We consider two types of variants (which are simulated separately): one with the same severity *p* as before (variant 1, orange) and a milder one with higher *p* (variant 2, purple).

First, we consider a scenario in which immunity only provides protection against symptoms, $\epsilon_{s}=0.4$ (Fig. 4A–C). In this case, protection against symptoms allows new variants to spread faster by increasing the amount of asymptomatic infections, resulting in larger outbreaks (Fig. 4B). Although the milder (purple) variant exhibits a faster epidemic growth rate and reaches a higher peak (Fig. 4B), it reaches similar peak fatality as the more severe (orange) variant (Fig. 4C). The asymptomaticity–fatality curve provides additional insight (Fig. 4A): even though a milder, invading variant (purple square) gives higher peak fatality than the original, wildtype variant (black circle), it leads to lower fatalities overall because deaths are concentrated over a shorter period of time in the epidemic; the relatively severe second-wave variant causes more deaths than the wildtype first wave, despite causing many fewer cases. In general, when δ is large, invading variants with similar asymptomaticity *p* will spread more effectively and result in worse population-level outcomes if immunity (either from vaccination or natural infection) provides protection against symptoms but not against infection or transmission.

Next, we consider a more realistic scenario in which immunity provides protection against both symptoms, $\epsilon_{s}=0.4$, and infection, $\epsilon_{i}=0.4$ (Fig. 4D–F). In this case, cross-protection against infection has a large effect on the more severe (orange) variant, causing its peak infection prevalence (Fig. 4E) and fatality (Fig. 4F) to be lower than that of the original, wildtype variant. Across a wide range of asymptomatic proportion *p*, we find that this immunity profile is sufficient to prevent worse outcomes at the population level; we note that the second wave of deaths is still high (and having higher peaks in some cases) even if the overall deaths are lower.

The outcomes in our simulations of invading variants resemble the dynamics of the SARS-CoV-2 Omicron variant. Despite moderate levels of vaccine effectiveness against symptomatic and reduced levels of severe cases caused by the Omicron variant, especially after booster shots (28), both vaccine- and infection-derived immunity provided limited protection against infections (29). This immune evasion helped the Omicron variant to cause more infections in South Africa than previous variants (30). Moreover, even though the Omicron variant is probably milder than the Delta variant (31, 32), the number of hospitalizations and deaths caused by the Omicron variant was higher than those caused by the Delta variant in many locations (33–35).

There are several limitations to our analysis. First, while we are able to generalize the model to include both presymptomatic and asymptomatic transmission, behavioral and intervention effects must be relatively large in order for the fatality to peak at intermediate levels of asymptomaticity (typically requiring a reduction in transmission rate of 60% or more for most of our chosen parameter sets). Second, the model framework is able to incorporate the impacts of immunity of infection, symptoms, and severity, but we neglected additional specific effects of immunity on transmission, which can also have important effects on disease dynamics (36, 37). In particular, if immunity provides stronger protection against transmission among immune individuals, population-level outcomes will be better than what our model predicts. Estimating protection against different endpoints (e.g., infection, symptom, death, and transmission) can help narrow this uncertainty. Finally, we assumed that asymptomatic and symptomatic individuals are infected for the same amount of time. Analysis of viral load trajectories suggests that asymptomatic individuals may clear infections faster (14); however, asymptomatic individuals may still transmit for a longer period of time if symptomatic individuals self-isolate quickly after symptom onset. The individual-level differences in the asymptomatic and symptomatic transmission time scale can have important implications for the inferences and predictions of pathogen dynamics (38, 39); nonetheless, we expect that predictions on the final size of the epidemic and total fatalities will be robust to small differences in the transmission time scale between asymptomatic and symptomatic individuals.

Even though we assumed a homogeneous population here, our analysis also has important implications for age-dependent heterogeneity in asymptomaticity (as shown in Fig. 1D). For example, vaccinations and intervention measures primarily targeting older individuals can prevent severe infections and improve individual-level outcomes. However, asymptomatic individuals, especially younger individuals with high contact rates, can still transmit to other, older individuals, potentially making population-level outcomes worse than they would be if intervention measures were distributed differently. We note that other factors, such as the efficacy of a vaccine and types of immunity provided by the vaccine, also play critical roles in making these decisions—in many cases, protecting the most vulnerable will be the optimal decision to minimize deaths (40).

The main conclusion of our analysis relies on having high levels of transmission reduction among symptomatic individuals (δ). For the simple model, the value of δ is limited by the amount of presymptomatic transmission, which makes high δ values unrealistic for COVID-19. Therefore, we extended our model to include both presymptomatic and asymptomatic transmission to show that our results hold for a more general case: for high levels of transmission reduction after symptom onset ($\delta_{s}$), epidemic fatalities peak at intermediate values of nonsymptomatic transmission. Based on early estimates for the contact tracing effectiveness (26) high levels are $\delta_{s}$ are plausible, but uncertainty remains. Furthermore, values of δ (and likewise, $\delta_{s}$) likely change over the course of an epidemic. During the exponential growth phase, δ is likely low because there is limited awareness for the outbreak and intervention measures in place. As the number of cases, hospitalizations, and fatalities increase, δ will also increase, reflecting changes in awareness-driven behavior and intensity of nonpharmaceutical interventions (41). Further analysis is needed to constrain the uncertainty in δ and to apply the model framework in distinct disease, policy, and socioeconomic contexts.

Via theory and simulation analysis of a series of simplified models, we have shown that asymptomatic infections (or, more generally, nonsymptomatic transmission) can under some conditions lead to a better outcome for many individuals while facilitating onward transmission that leads to a worse outcome for the population as a whole. Extending our framework further shows that immunity profile (i.e., reduction of infection, symptoms, and/or severity due to immunity) plays a critical role in determining the dynamics of future variants; these results extend previous work on postpandemic trajectories that focus primarily on cross-immunity (42, 43). For example, while protection against symptoms protects health at the individual level, it can lead to more infections, and potentially more deaths, at the population level. A similar concern was raised in prioritizing vaccine choices that could reduce severe outcomes vs. others that could reduce transmission (9). Our conclusions echo earlier findings by (44), who showed that vaccine-derived immunity against diseases can promote the evolution of more virulent strains in unvaccinated individuals. By allowing explicit parasite evolution, they also showed that total malaria mortality peaks at intermediate levels of vaccination; our analysis reveals that the differences in symptomatic and asymptomatic transmission behavior can give rise to a similar effect even in the absence of evolution.

Our work uses theoretical models to illustrate the potential for asymptomatic infection and transmission to make population-level outcomes worse. Applying these ideas to specific outbreak scenarios will require narrowing down uncertainties in key parameters, such as the degree of symptom-responsive transmission reduction and immunity profiles. For example, we simulated unmitigated outbreaks with fixed parameters but epidemic dynamics, especially those of SARS-CoV-2, are more complex, reflecting changes in intervention efforts and the emergence of new variants. Calibrating the model to outbreak data is therefore critical to understanding the role of asymptomatic transmission across different epidemic phases. We also showed that asymptomatic infections can have important implications for evolutionary dynamics, but their contributions in driving evolutionary dynamics of SARS-CoV-2 is yet unclear. Our study provides a starting point for exploring these questions in more detail.

As is increasingly evident, SARS-CoV-2 has proven hard to control in large part because transmission is often decoupled from symptoms. Our model reinforces the need for dual approaches—prioritizing the reduction of asymptomatic spread (e.g., via risk awareness campaigns (45–47), asymptomatic testing programs (48–50), mask-wearing indoors and in crowded environments (51–53), and through improvements in ventilation (54, 55)) while also improving the treatment of symptomatic cases, particularly amongst older individuals at highest risk for severe outcomes. Given the link between age and asymptomatic infections (15), interventions may consider different approaches in strongly age-structured populations (e.g., schools or long-term care facilities). Mass vaccination is also expected to be important especially if future vaccines induce more transmission blocking. As more variants continue to emerge, monitoring the impacts of preexisting immunity (whether through vaccination and/or infections (56)) on preventing infections, and not just disease, will be critical to controlling the course of this and future pandemics.

## Materials and methods

### Epidemic models with asymptomatic infection and transmission in the absence of immunity

We consider a compartmental model with asymptomatic and symptomatic infections in a homogeneously mixing population. The basic model dynamics are as follows:


(3) $$\dot{S}=-\beta_{a}SI_{a}-(1-\delta )\beta_{s}SI_{s}$$

(4) $$\dot{E}=\beta_{a}SI_{a}+(1-\delta )\beta_{s}SI_{s}-\nu E$$

(5) $${\dot{I}}_{a}=p\nu E-\gamma_{a}I_{a}$$

(6) $${\dot{I}}_{s}=(1-p)\nu E-\gamma_{s}I_{s}$$

(7) $$\dot{R}=\gamma_{a}I_{a}+(1-f)\gamma_{s}I_{s}$$

(8) $$\dot{D}=f\gamma_{s}I_{s}$$

where the transmission rate β and removal rate γ can be potentially differ between asymptomatic and symptomatic individuals. Similar models have been previously used to study the dynamics of SARS-CoV-2 (57, 17, 39). Here, δ denotes the reduction in transmissibility due to responsive measures taken by symptomatic individuals. Throughout the paper, we use parameters that are broadly consistent with the dynamics of the originating strain of SARS-CoV-2: $\beta_{s}=0.8/\mathrm{day}$, $\beta_{a}=0.75\beta_{s}$, 1/ν=2days, $1/\gamma_{s}=1/\gamma_{a}=5\mathrm{days}$, and f=0.01 (58). Under this parameterization, we have symptomatic and asymptomatic reproduction numbers of $R_{s}=4$ and $R_{a}=3$.

We then extend this model to include both presymptomatic and asymptomatic transmission. This is done by adding a presymptomatic compartment before entering symptomatic or asymptomatic compartments (17).


(9) $$\dot{S}=-\beta_{p}SI_{p}-\beta_{a}SI_{a}-(1-\delta )\beta_{s}SI_{s}$$

(10) $$\dot{E}=\beta_{a}SI_{a}+\beta_{p}SI_{p}+(1-\delta_{s})\beta_{s}SI_{s}-\nu E$$

(11) $${\dot{I}}_{p}=\nu E-\sigma I_{p}$$

(12) $${\dot{I}}_{a}=p\sigma I_{p}-\gamma_{a}I_{a}$$

(13) $${\dot{I}}_{s}=(1-p)\sigma I_{p}-\gamma_{s}I_{s}$$

(14) $$\dot{R}=\gamma_{a}I_{a}+(1-f)\gamma_{s}I_{s}$$

(15) $$\dot{D}=f\gamma_{s}I_{s}$$

For this generalized model, the presymptomatic $R_{p}$, symptomatic $R_{s}$, and asymptomatic $R_{a}$ reproduction numbers are given by $R_{p}=\beta_{p}/\sigma$, $R_{s}=\beta_{s}/\gamma_{s}$, and $R_{a}=\beta_{a}/\gamma_{a}$ in the absence of the behavioral effect; these reproduction numbers represent the average number of secondary cases caused by an infected individual in each compartment. Then, the reproduction number of individuals who will eventually develop symptoms is equal to: $R_{p}+R_{s}$; similarly, the reproduction number of individuals who remain asymptomatic is equal to: $R_{p}+R_{a}$. Since the proportion *p* of all infections is asymptomatic, the basic reproduction number is given by the weighted average of these two reproduction numbers:


(16) $$R_{0}=p(R_{p}+R_{a})+(1-p)(R_{p}+R_{s})=R_{p}+pR_{a}+(1-p)R_{s}.$$

Then, the proportion of nonsymptomatic transmission *ϕ* is given by:


(17) $$\phi =\frac{R_{p}+pR_{a}}{R_{0}}.$$

For simulations of the combined model, we start by fixing the reproduction number of individuals who will eventually develop symptoms: $R_{\mathrm{symp}}=R_{p}+R_{s}=4$. Consistent with previous assumptions, we also assume that asymptomatic reproduction number is lower than that of the symptomatic reproduction number: $R_{a}=\rho R_{s}$, where ρ=0.75. Then, for a given value of the proportion of nonsymptomatic transmission *ϕ* and proportion of nonsymptomatic transmission caused by the presymptomatic transmission, $\eta =R_{p}/(R_{p}+pR_{a})$, we can solve for the transmission rate for each compartment β and the proportion asymptomatic *p*. More specifically:


(18) $$R_{p}=\frac{R_{\mathrm{symp}}}{1+y}$$

(19) $$R_{s}=R_{\mathrm{symp}}-R_{p}$$

(20) $$R_{a}=\rho R_{s}$$

(21) $$p=\left(\frac{1}{\eta }-1\right)\frac{R_{p}}{R_{a}},$$

where y=(1/ϕ−1)/η+(1/η−1)/ρ. In order to keep the mean infectious period fixed, we assume 1/σ=2days and $1/\gamma_{s}=1/\gamma_{a}=3\;\mathrm{days}$. All other parameters are same as before.

### Epidemic models with asymptomatic infection and transmission in the presence of immunity

We model the spread of infection in a partially immune population by assuming a leaky protection. The leaky assumption has been widely used throughout the SARS-CoV-2 pandemic (36). Another option is to rely on the all-or-nothing assumption (59–61); for simplicity, we do not explore this option. Then, the model equations are given by:


(22) $$\dot{S}=-\lambda (t)S$$

(23) $$\dot{E}=\lambda (t)S-\nu E$$

(24) $${\dot{I}}_{a}=p\nu E-\gamma_{a}I_{a}$$

(25) $${\dot{I}}_{s}=(1-p)\nu E-\gamma_{s}I_{s}$$

(26) $$\dot{R}=\gamma_{a}I_{a}+(1-f)\gamma_{s}I_{s}$$

(27) $$\dot{D}=f\gamma_{s}I_{s}$$

(28) $${\dot{S}}_{p}=-(1-\epsilon_{i})\lambda (t)S_{p}$$

(29) $${\dot{E}}_{p}=(1-\epsilon_{i})\lambda (t)S_{p}-\nu E_{p}$$

(30) $${\dot{I}}_{\;p,a}=(1-(1-\epsilon_{s})(1-p))\nu E_{p}-\gamma_{a}I_{\;p,a}$$

(31) $${\dot{I}}_{\;p,s}=(1-\epsilon_{s})(1-p)\nu E_{p}-\gamma_{s}I_{\;p,s}$$

(32) $${\dot{R}}_{p}=\gamma_{a}I_{\;p,a}+(1-(1-\epsilon_{d})f)\gamma_{s}I_{\;p,s}$$

(33) $${\dot{D}}_{p}=(1-\epsilon_{d})f\gamma_{s}I_{\;p,s}$$

where $0\leq \epsilon_{i},\epsilon_{s},\epsilon_{d}\leq 1$ represents the degree of protection against infection, symptoms and death, respectively. The force of infection λ(t) is given by:


(34) $$\lambda (t)=\beta_{a}(I_{a}+I_{\;p,a})+(1-\delta )\beta_{s}(I_{s}+I_{\;p,s}).$$

Here, subscripts *p* denote individuals who are immune and therefore are protected, and as before the subscripts *a* and *s* denote asymptomatic and symptomatic infections.

## Supplementary Material

pgad106_Supplementary_Data

## Data Availability

All data and code are stored in a publicly available GitHub repository (https://github.com/parksw3/asymptomaticvariant).
