# Supplementary material for: Intermediate levels of asymptomatic transmission can lead to the highest epidemic fatalities
Source: PNAS Nexus. 2023 Mar 29;2(4):pgad106. doi: 10.1093/pnasnexus/pgad106 (PMC10118396; doi:10.1093/pnasnexus/pgad106)
Supplement: pgad106_Supplementary_Data [file pgad106_supplementary_data.pdf]

Supplementary Materials for Intermediate levels of asymptomatic transmission can lead to the highest epidemic fatalities

Sang Woo Park[1], Jonathan Dushoff[2,3,4], Bryan T. Grenfell[1,5], Joshua S. Weitz[6,7,8,*]

1 Department of Ecology and Evolutionary Biology, Princeton University, Princeton, NJ, USA
2 Department of Biology, McMaster University, Hamilton, ON, Canada
3 Department of Mathematics and Statistics, McMaster University, Hamilton, ON, Canada
4 M. G. DeGroote Institute for Infectious Disease Research, McMaster University, Hamilton, ON, Canada
5 Princeton School of Public and International Affairs, Princeton University, Princeton, NJ, USA
6 School of Biological Sciences, Georgia Institute of Technology, Atlanta, GA, USA
7 School of Physics, Georgia Institute of Technology, Atlanta, GA, USA
8 Institut de Biologie, École Normale Supérieure, Paris, France

*Corresponding author: jsweitz@gatech.edu

Supplementary Tables

Parameter	Description	Assumed values
β_s	Symptomatic transmission rate	0.8/days
β_a	Asymptomatic transmission rate	$0.75\beta_s$
$1/\nu$	Mean latent period	2 days
$1/\gamma_s$	Mean symptomatic infectious period	5 days
$1/\gamma_a$	Mean asymptomatic infectious period	5 days
p	Proportion asymptomatic	0–1
f	Fatality rate for symptomatic case	0.01
δ	Reduction in symptomatic transmission rate	0–1

Table S1: Parameter descriptions and values for the basic asymptomatic model.

**Parameter**	**Description**	**Assumed values**
β_s	Symptomatic transmission rate	See Materials and Methods
β_a	Asymptomatic transmission rate	See Materials and Methods
β_p	Presymptomatic transmission rate	See Materials and Methods
$1/\nu$	Mean latent period	2 days
$1/\sigma$	Mean presymptomatic infectious period	2 days
$1/\gamma_s$	Mean symptomatic infectious period	3 days
$1/\gamma_a$	Mean asymptomatic infectious period	3 days
p	Proportion asymptomatic	0–1
f	Fatality rate for symptomatic case	0.01
δ_s	Reduction in symptomatic transmission rate	0–1

Table S2: Parameter descriptions and values for the generalized asymptomatic model.

Parameter	Description	Assumed values
β_s	Symptomatic transmission rate	0.8/days
β_a	Asymptomatic transmission rate	$0.75\beta_s$
$1/\nu$	Mean latent period	2 days
$1/\gamma_s$	Mean symptomatic infectious period	5 days
$1/\gamma_a$	Mean asymptomatic infectious period	5 days
p	Proportion asymptomatic	0–1
f	Fatality rate for symptomatic case	0.01
δ	Reduction in symptomatic transmission rate	0–1
ϵ_i	Protection against infection	0–0.8
ϵ_s	Protection against symptoms	0–0.8
ϵ_d	Protection against deaths	0–0.8

Table S3: Parameter descriptions and values for the asymptomatic model with immunity.

Supplementary Figures

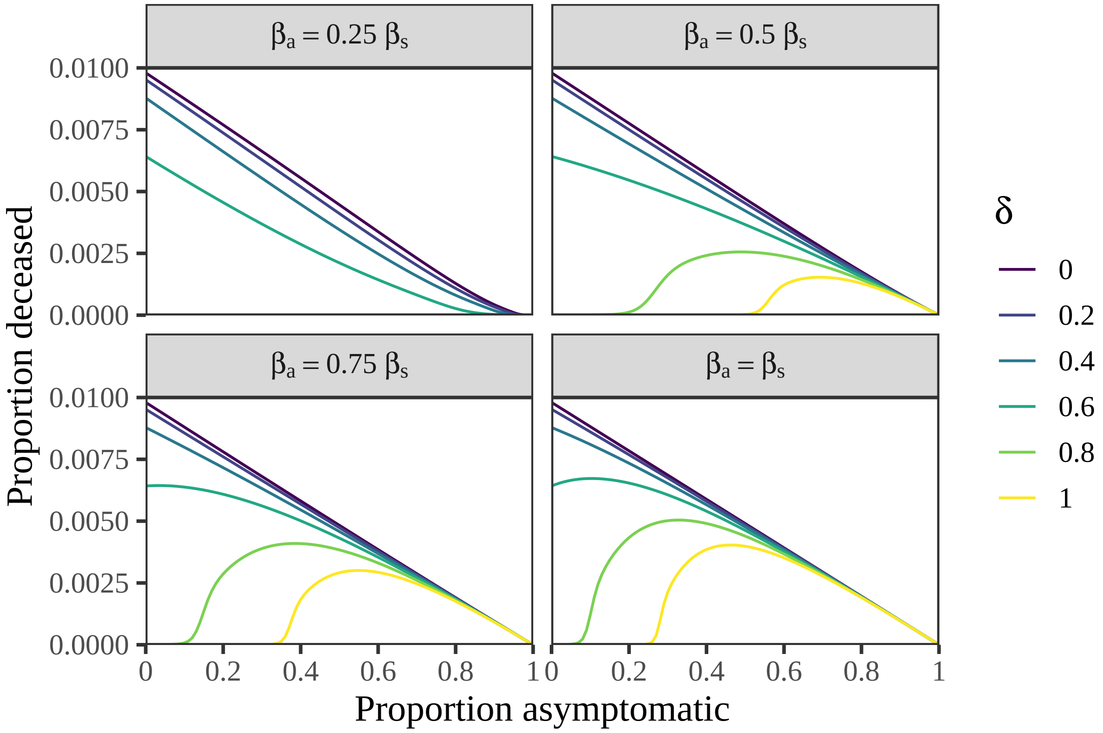

Figure S1: **Simulations of a model with asymptomatic transmission and symptom-responsive transmission reduction for a wide range of asymptomatic transmissibility.** Total deaths as a function of the proportion of asymptomatic infections p across a wide range scenarios for δ. We simulate the model for 365 days, assuming $\beta_s = 0.8$/day, $\nu = 0.5$/day, $\gamma_s = \gamma_a = 0.2$/day, and $f = 0.01$, and an initial exposed proportion of 10^{-4}. We allow the ratios between the asymptomatic transmission rate β_a and symptomatic transmission rate β_s to vary between 0.25 and 1. See Materials and Methods for model details and Supplementary Table S1 for parameter descriptions and values.

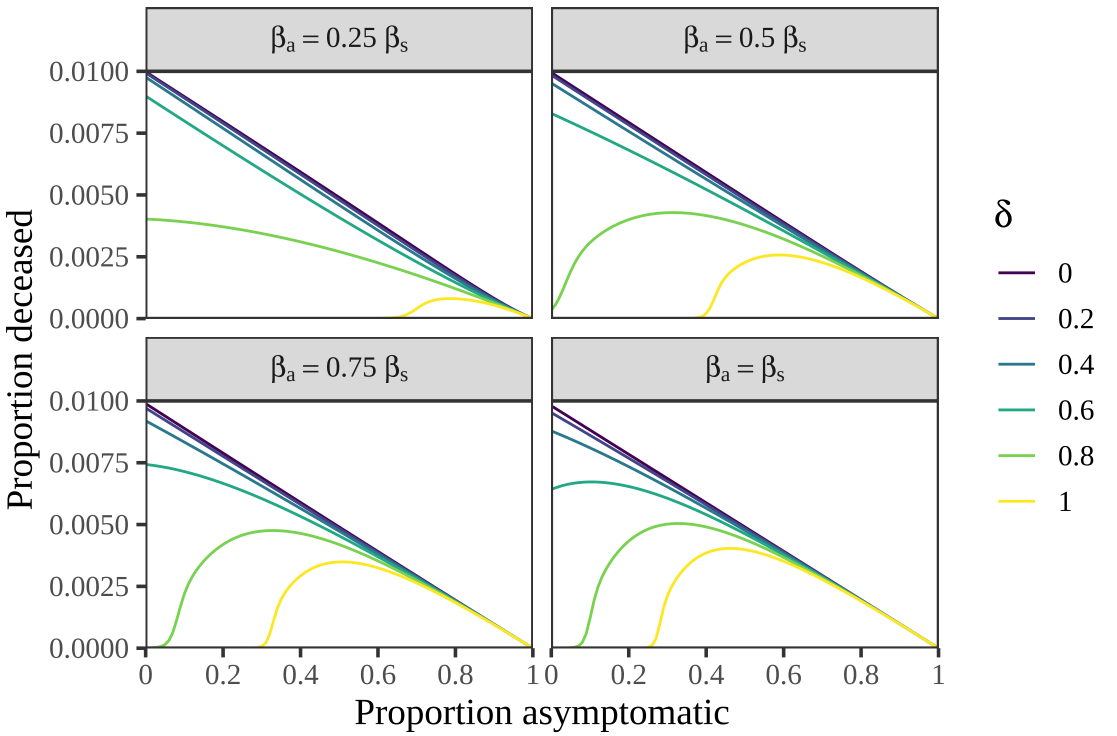

Figure S2: **Simulations of a model with asymptomatic transmission and symptom-responsive transmission reduction for a wide range of asymptomatic transmissibility and a fixed $\mathcal{R}_0$ value at intermediate asymptomaticity.** Total deaths as a function of the proportion of asymptomatic infections p across a wide range scenarios for δ. We simulate the model for 365 days, assuming $\nu = 0.5$/day, $\gamma_s = \gamma_a = 0.2$/day, and $f = 0.01$, and an initial exposed proportion of 10^{-4}. We allow the ratios between the asymptomatic transmission rate β_a and symptomatic transmission rate β_s to vary between 0.25 and 1. We also fix the basic reproduction number $\mathcal{R}_0 = 4$ when there are intermediate levels of asymptomaticity $p = 0.5$ and no reduction in symptomatic transmission rate $\delta = 0$. See Materials and Methods for model details and Supplementary Table S1 for parameter descriptions and values.

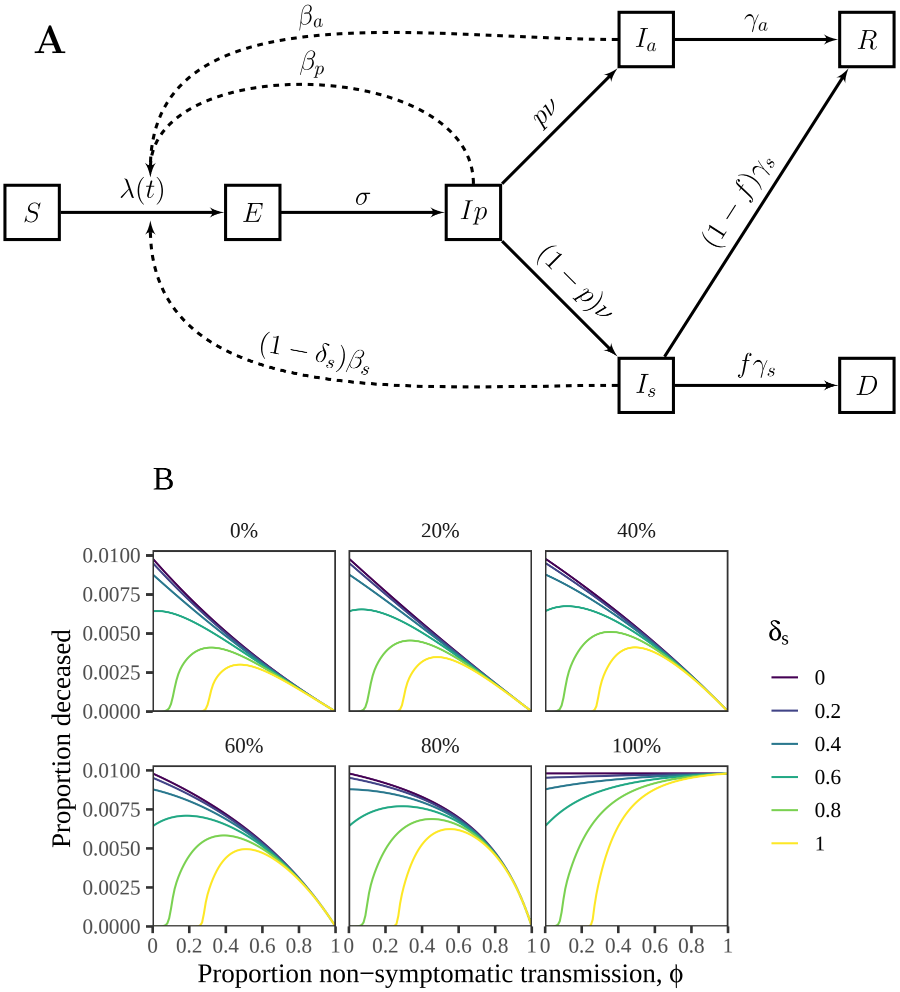

Figure S3: **Schematic diagram and simulations of a model with pre-symptomatic and asymptomatic transmission and symptom-responsive transmission reduction.** (A) S represents susceptible individuals; E represents exposed individuals; I_p represents pre-symptomatic individuals; I_a represents symptomatic individuals; I_s represents symptomatic individuals; R represents recovered individuals; and D represents deceased individuals. See Methods for model details. (B) Total deaths as a function of the proportion of non-symptomatic transmission ϕ across a wide range scenarios for δ_s and proportion of non-symptomatic transmission caused by the pre-symptomatic transmission, η (between 0% and 100%). See Materials and Methods for model details and Supplementary Table S2 for parameter descriptions and values.
